# Down-Regulation of Nicotinamide N-methyltransferase Induces Apoptosis in Human Breast Cancer Cells via the Mitochondria-Mediated Pathway

**DOI:** 10.1371/journal.pone.0089202

**Published:** 2014-02-18

**Authors:** Jun Zhang, Yanzhong Wang, Guiling Li, Haitao Yu, Xinyou Xie

**Affiliations:** 1 Clinical Laboratory, Sir RunRun Shaw Hospital, School of Medicine, Zhejiang University, Hangzhou, China; 2 Key Laboratory of Biotherapy of Zhejiang Province, Hangzhou, China; Texas Tech University Health Sciences Center, United States of America

## Abstract

Nicotinamide N-methyltransferase (NNMT) has been found involved in cell proliferation of several malignancies. However, the functional role of NNMT in breast cancer has not been elucidated. In the present study, we showed that NNMT was selectively expressed in some breast cancer cell lines, down-regulation of NNMT expression in Bcap-37 and MDA-MB-231 cell lines by NNMT shRNA significantly inhibited cell growth *in vitro*, decreased tumorigenicity in mice and induced apoptosis. The silencing reciprocal effect of NNMT was confirmed by over-expressing NNMT in the MCF-7 and SK-BR-3 breast cancer cell lines which lack constitutive expression of NNMT. In addition, down-regulation of NNMT expression resulted in reducing expression of Bcl-2 and Bcl-xL, up-regulation of Bax, Puma, cleaved caspase-9, cleaved caspase-3 and cleaved PARP, increasing reactive oxygen species production and release of cytochrome c from mitochondria, and decreasing the phosphorylation of Akt and ERK1/2. These data suggest that down-regulation of NNMT induces apoptosis via the mitochondria-mediated pathway in breast cancer cells.

## Introduction

Breast cancer is one of the most common causes of cancer-related death in women, which accounts for one in four cancer-related deaths in the United States [1]. In China, according to the most updated but limited cancer registries, breast cancer is the fifth leading cause of cancer-related death for females [2]. There is a decline in breast cancer mortality since 1995 [1]–[3], however, breast cancer is far from being cured because of delayed detection, the progressive growth, late detection of metastases and resistant to conventional therapies. Therefore, there is an urgent need to identify new biomarkers, which are warranted to provide more information on the tumor biology, chemotherapeutic effects, allowing a better prognostic and possibly predictive stratification of patients. Recent researches have reported that the growth of tumor cells may be inhibited via the mitochondrial apoptotic pathway in breast cancer [4], [5].

Nicotinamide N-methyltransferase (NNMT, EC 2.1.1.1), a cytoplasmic enzyme belonging to Phase IIMetabolizing Enzymes, which catalyzes the methylation of nicotinamide and other pyridines to form pyridinium ions using S-adenosyl-L-methionine as methyl donor [6]. NNMT also plays a vital role in nicotinamide metabolism and the biotransformation of many drug and other xenobiotic compounds [7]. NNMT exhibits a high expression in the liver and follows a bimodal frequency distribution which might result in differences among individuals in the metabolism and therapeutic effect of drugs [8]. Proteomics analysis and DNA microarray analysis showed that NNMT was expressed at markedly higher levels in several kinds of cancers. It was identified as a novel serum tumor marker for colorectal cancer (CRC) in 2005 [9]. In addition to CRC, abnormal expression of NNMT was also reported in other tumors such as papillary thyroid cancer [10], glioblastoma [11], gastric cancer [12], [13], renal carcinoma [14]–[16], oral squamous cell carcinoma [17], [18], lung cancer [19], pancreatic cancer [20], [21] and ovarian clear cell carcinoma [22]. Our previous studies have also shown that NNMT is over-expressed in a large proportion of renal cell cancers and that high expression of NNMT is significantly associated with unfavorable prognosis [23]. The most recent studies showed that knockdown of NNMT was able to inhibit the proliferation of KB cancer cells [24], renal carcinoma cells [25] and oral cancer cells [26], and NNMT expression was involved in maintaining cell proliferation by increasing the activity of Complex I (NADH:ubiquinone oxidoreductase) in SH-SY5Y neuroblastoma cells [27]. However, the mechanism of NNMT in cell proliferation is largely unknown and the functional role of NNMT in breast cancer has not been reported.

In the present study, we investigated the expression of NNMT in human breast cancer cell lines and found that shRNA targeted against NNMT significantly decreased cell growth, inhibited tumorigenicity in mice and induced apoptosis via the mitochondria-mediated pathway.

## Materials and Methods

### Ethics statement

All experiments in the present study were conducted in strict accordance with the recommendations in the Guide for the Care and Use of Laboratory Animals published by the US National Institutes of Health. The animal experiments were previously approved by the Animal Care and Use Committee at Sir RunRun Shaw hospital of Zhejiang University (Permit Number: 20120222-31). The number of animals used was minimized, and all necessary precautions were taken to mitigate pain or suffering.

### Cell Lines, culture conditions, and antibodies

The human breast cancer cell lines Bcap-37, MCF-7, MCF-7/ADR, SK-BR-3, MDA-MB-468 and MDA-MB-231 were obtained from the Cell Bank at the Chinese Academy of Sciences (Shanghai, China). MCF-7/ADR was developed from the parental MCF-7 cells by stepwise selection for resistance with increasing concentration of doxorubicin in different labs and cultured in RPMI-1640 medium (Gibco, Grand Island, NY, USA) with 1 µM doxorubicin (Sigma, St. Louis, MO). Bcap-37, MCF-7, SK-BR-3, MDA-MB-468 and MDA-MB-231 cells were cultured in DMEM medium (Gibco, Grand Island, NY, USA). All media were supplemented with 10% fetal bovine serum (Gibco, Long Island, NY,USA), 100 U/ml of penicillin (Sigma, St. Louis, MO, USA) and 100 µg/ml of streptomycin (Sigma, St. Louis, MO, USA), and cells were maintained at 37°C in a humidified 5% CO_2_ incubator.

The following antibodies were obtained from Cell Signaling Technology (Beverly, Massachusetts, USA): anti-Bcl-2, anti-Bax, anti-Bcl-xL, anti-Puma, anti-cytochrome c, anti-caspase-3, anti-cleaved caspase-3, anti-caspase-9, anti-cleaved caspase-9, anti-PARP, anti-cleaved-PARP, anti-Akt, anti-p-Akt(Ser473), anti-ERK1/2, anti-p-ERK1/2 (Thr202/Tyr204) and anti-GAPDH. Mouse anti-NNMT monoclonal antibody 1E7 was prepared by hybridoma technique as previously described [23]. Goat anti-mouse and goat anti-rabbit HRP-conjugated antibodies were obtained from Zhongshan Goldenbridge Biotechnology Co. (Beijing, China).

### RNA isolation and real-time quantitative RT-PCR

Differential Bcl-2 family members and NNMT gene expression levels in breast cancer cells were assessed by real-time quantitative RT-PCR analysis, using the SYBR Premix EX Taq™ RealTime PCR Detection System (TaKaRa Biotechnology, Dalian, China). RNA was isolated with TRIZOL reagent (Invitrogen, Carlsbad, CA, USA) and reverse transcribed into cDNAs with the M-MLV Reverse Transcriptase kit (Promega, Madison, WI, USA). PCR primer sequences used are listed in Table 1. The experiments were run as follows: an initial denaturation step of 95°C for 30 seconds, followed by 40 cycles of 95°C for 5 seconds and 60°C for 34 seconds using an ABI PRISM 7500 Fast Real-Time PCR System. All the experiments were independent and conducted at least three times. The results were calculated using 2^−ΔΔCt^ method. The data were normalized to GAPDH and then compared to the control group, which was normalized as 1.

**Table 1:** Primer series of NNMT, Bcl-2, Bax, Bcl-xL, Puma and GAPDH gene.

Gene	Forward sequence	Reverse sequence
NNMT	5′-GAATCAGGCTTCACCTCCAA-3′	5′-CCCAGGAGATTATGAAACACC-3′
Bcl-2	5′-CGGGAGATGTCGCCCCTGGT-3′	5′-GCATGCTGGGGCCGTACAGT-3′
Bax	5′-GGCCGGGTTGTCGCCCTTTT-3′	5′-CCGCTCCCGGAGGAAGTCCA-3′
Bcl-xL	5′-CGGTACCGGCGGGCATTCAG-3′	5′-CGGCTCTCGGCTGCTGCATT-3′
Puma	5′-TGGGGTCTGCCCAGGCATGT-3′	5′-GAGCTGCCCTCCTGGCGTG-3′
GAPDH	5′-ACGGATTTGGTCGTATTGGG-3′	5′-CCTGGAAGATGGTGATGGGATT-3′

### Western blot analysis

Cell extracts were prepared with RIPA lysis buffer (Beyotime biotechnology, Shanghai, China). Protein concentrations were measured by a BCA Protein Assay Kit (Beyotime biotechnology, Shanghai, China) using bovine serum albumin (BSA) as the standard. A total of 50 µg of protein samples from each cell line was subjected to 10% sodiumdodecyl sulfate-polyacrylamide gel electrophoresis (SDS-PAGE) and transferred to Immobilon P Transfer Membrane (Millipore, Bedford, MA, USA). After regular blocking and washing, the membranes were incubated with primary antibodies overnight at 4°C, followed by incubating with HRP-conjugated secondary antibodies for 1 h at room temperature. Signals were visualized using enhanced chemiluminescence detection reagents (Millipore, Billerica, MA, USA) and imaged using an Image Quant LAS-4000 (Fujifilm, Tokyo, Japan). All the experiments were independent and conducted at least three times. The protein quantification of the Western blot results were normalized to GAPDH and then compared to the control group, which was normalized as 1.

### Lentiviral shRNA-NNMT infection

Lentiviral vectors against NNMT were synthesized by GeneChem Co. Ltd (Shanghai, China). Table 2 showed the sequences of NNMT shRNA 1#, NNMT shRNA 2#, shRNA NC and the frame of lentiviral vectors. Bcap-37 and MDA-MB-231 cells were infected with lentivirus containing shRNA (NNMT shRNA 1#, NNMT shRNA 2#, shRNA NC; MOI = 100 for Bcap-37, MOI = 10 for MDA-MB-231) after seeded (2×10^5^ cells/well) in six-well plates for 24 h. Ten hours after co-culturing with lentivirus, the supernatant was replaced with fresh medium. 48 h after infection, the transduced cells were sorted for GFP-positive cell populations by BD FACS Aria II System (BD Biosciences, San Jose, CA, USA) and then subjected to functional assays. The efficiency of gene silencing was detected by real-time RT-PCR and Western blot analysis as described above. Cells infected with shRNA NC were used as negative control.

**Table 2:** Construction frame of lentiviral vectors against NNMT.

Lentiviral vector	5′	STEM	Loop	STEM	3′
NNMT shRNA 1#	T	GCTCAAGAGCAGCTACTACAT	CTCGAG	ATGTAGTAGCTGCTCTTGAGC	TTTTTTC
	TCGAGAAAAAA	GCTCAAGAGCAGCTACTACAT	CTCGAG	ATGTAGTAGCTGCTCTTGAGC	A
NNMT shRNA 2#	T	ACCCTCGGGATTACCTAGAAA	CTCGAG	TTTCTAGGTAATCCCGAGGGT	TTTTTTC
	TCGAGAAAAAA	ACCCTCGGGATTACCTAGAAA	CTCGAG	TTTCTAGGTAATCCCGAGGGT	A
shRNA NC	T	TTCTCCGAACGTGTCACGT	CTCGAG	ACGTGACACGTTCGGAGAA	TTTTTTC
	TCGAGAAAAAA	TTCTCCGAACGTGTCACGT	CTCGAG	ACGTGACACGTTCGGAGAA	A

### siRNA transfection

When detecting the ROS production, we chose specific siRNAs to silence NNMT expression in order to avoid the fluorescence of GFP in lentiviral vector interfering with the ROS measurement. 2×10^5^ cells (Bcap-37 and MDA-MB-231) were plated in 6-well plates in 2 ml antibiotic-free DMEM medium supplemented with FBS and 8 µl the NNMT specific siRNAs (10 µM) (sc-61213, Santa Cruz Biotechnology, CA, USA) were transfected into cultured cells at a final concentration of 80 nM using Lipofectamine 2000 transfection reagent (Invitrogen Life Technologies, Carlsbad, CA, USA) according to the manufacturer’s instructions. Control siRNA contained a scrambled sequence that would not lead to the specific degradation of any known cellular mRNA.

### NNMT plasmids transfection and stable cell strains selection

The complementary DNA of NNMT gene from pGEX-4T-2/NNMT plasmids [23] was amplified by PCR using specific primers (forward 5′-GAATCAGGCTTCACCTCCAA-3′ and reverse 5′-TCACACCGTCTAGGCAGAAT-3′) and cloned into the pcDNA3.1 plasmid of which digested with the restriction enzyme BamHI/XhoI (named as pcDNA3.1/NNMT). MCF-7 and SK-BR-3 cells were transfected with pcDNA3.1/NNMT or pcDNA3.1 vector using Lipofectamine^TM^2000 according to the manufacturer’s instructions and then cells were grown in complete medium containing 800 mg/L of geneticin (G418; Gibco, Grand Island, NY, USA). After G418 selection for 2 weeks, single colonies were picked into 96-well plates to proliferate separately and evaluated for NNMT expression by Real-Time quantitative RT-PCR and Western blotting as described above. Two monoclonal cell strains with stable NNMT overexpression, MCF-7/NNMT-1 and MCF-7/NNMT-2; SK-BR-3/NNMT-1 and SK-BR-3/NNMT-2, were selected for further analysis. One monoclonal cell strain with pcDNA3.1 transfection named as MCF-7/Vector or SK-BR-3/Vector was used as control.

### MTT assay

Cell growth was assessed by the colorimetric MTT assay. All the cells were prepared at a concentration of 2×10^4^ cells/ml. Aliquots (200 µL) were dispensed into 96-well flat-bottom plates. The cells were allowed to attach for five hours at 37°C and 5% CO_2_, and cell growth was evaluated for up to 120 h. Subsequently, 20 µl of the 5 g/L MTT solution (Sigma, St. Louis, MO, USA) was added to each well. After incubation for 4 h at 37°C, the supernatant was removed carefully, and 150 µl of dimethyl sulfoxide (DMSO, Sigma, St. Louis, MO, USA) was added to each well. Ten minutes after incubation at 37°C, the absorbance value of each well was read at 490 nm using an ELISA plate reader instrument (Model 680, BIO RAD, Osaka, Japan). All experiments were repeated at least three times. The absorbance values at each time point were compared to that of control group at 0 h, which was normalized as 100%.

### Plate colony formation assay

The ability of cells to form macroscopic colonies was determined by a plate colony formation assay. Cells in the logarithmic phase were collected to prepare single cell suspensions and were seeded in six-well plates (Bcap-37∶400/well, MDA-MB-231∶1000/well, MCF-7∶400/well, SK-BR-3∶400/well). After incubation at 37°C for 14 d, colonies were rinsed with PBS, fixed with methanol at −20°C for 5 min, and stained with Giemsa (Sigma, St. Louis, MO, USA) for 20 min. Only clearly visible colonies (foci>50 µm) were counted, and cloning efficiency was calculated using following formula: Cloning efficiency = (number of clones/number of cells inoculated)×100%. Experiments were repeated at least three times.

### Soft agar colony formation assay

Soft agar clonogenic assays were performed at least three times to assess anchorage-independent growth. Cells (1×10^4^/well in six-well plates) were detached and plated in DMEM medium containing 0.3% low melting point (LMP) agar with a 0.5% LMP agar layer underlay. The cells were cultured at 37°C in 5% CO2, and every 3 days 500 µl of fresh medium was added to each well. The number of foci>100 µm was counted after 14 days.

### Xenograft experiments

Male specific pathogen-free BALB/c Nude mice (6 weeks old, 18–20 g body weight) were handled under pathogen-free sterile conditions, maintained on a 12 hour light:dark cycle (lights on at 7∶00am) with continuous access to sterile food and water. For tumorigenicity assays, 2×10^6^ cells each of the Bcap-37/NC, Bcap-37/NNMT shRNA 1# and Bcap-37/NNMT shRNA 2# cells were subcutaneously injected into the upper portion of the right hind limb of 6 BALB/c Nude mice for each group. Tumor size was measured using calipers every 3 days and calculated by the formula: 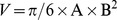, where V is volume, A is the largest diameter and B is the smallest diameter. With the exception of mice with large tumor burdens (A<20 mm, B<10 mm), animals were euthanized by Isoflurane inhalation (Abbot Laboratories Ltd., North Chicago, IL) then cervical dislocation was conducted 30 days after injection. At the end of experiment, tumors were harvested and weighed.

### Apoptosis analysis

Apoptosis was detected by flow cytometric analysis using an Annexin V-PE/7-AAD Apoptosis Detection Kit (MultiSciences Biotech Co., Ltd, Hangzhou, China) according to the protocol provided. Briefly, the cells were seeded (3×10^5^ cells/well) in a 6-well plate and incubated for 48 h. All groups were treated with staurosporine (Sigma, St. Louis, MO, USA) (2 µM for Bcap-37 cells, 100 nM for MDA-MB-231, 1 µM for MCF-7 and SK-BR-3), an inducer of apoptotis, as a positive control. Then, the treated cells were harvested and incubated with Annexin V-PE and 7-AAD for 15 min at room temperature in the dark and immediately analyzed by flow cytometry (FACScalibur flow cytometer, BD, CA, USA). Each experiment was carried out for at least three times.

### Measurement of ROS production by flow cytometry

Production of intracellular ROS was detected by flow cytometry using the fluorescent probe 2′,7′-dichlorodihydrofluorescein diacetate (DCFH-DA, Sigma, St. Louis, MO, USA). The cells were treated with 10 µM DCFH-DA for 30 min in the dark with a new medium after cultured in 60-mm dishes for 48 h, washed once with PBS, detached by trypsinization, collected by centrifugation, and suspended in PBS. The intracellular ROS, as indicated by the fluorescence of dichlorofluorescein (DCF), were measured by flow cytometry (FACScalibur flow cytometer, BD, CA, USA) at excitation and emission wavelengths of 485 nm and 530 nm, respectively. All the experiment were repeated at least three times. The results of the cells treated with NNMT siRNA were compared to that of the ones treated with control siRNA, which were normalized as 1.

### Cytochrome c release measurement

The mitochondria fraction and cytosolic fraction were isolated using cytochrome c releasing apoptosis assay kit (BioVision, Inc., Mountain View, CA, USA) according to the manufacturer’s instructions. The detection of Cyt c was performed by Western blot analysis as described above. All the experiments were independent and conducted at least three times. The protein quantification of the Western blot results were normalized to GAPDH and then compared to the NC group, which was normalized as 1.

### Statistical analysis

All statistical analyses were carried out using the SPSS 19.0 statistical software package. Data were presented as mean ± SD. The two-tailed independent-samples Student’s t-test was performed to analyze the difference among groups. Statistical significance was define as *p<0.05 and **p<0.01.

## Results

### Differential NNMT expression in breast cancer cell lines

**Figure 1:**
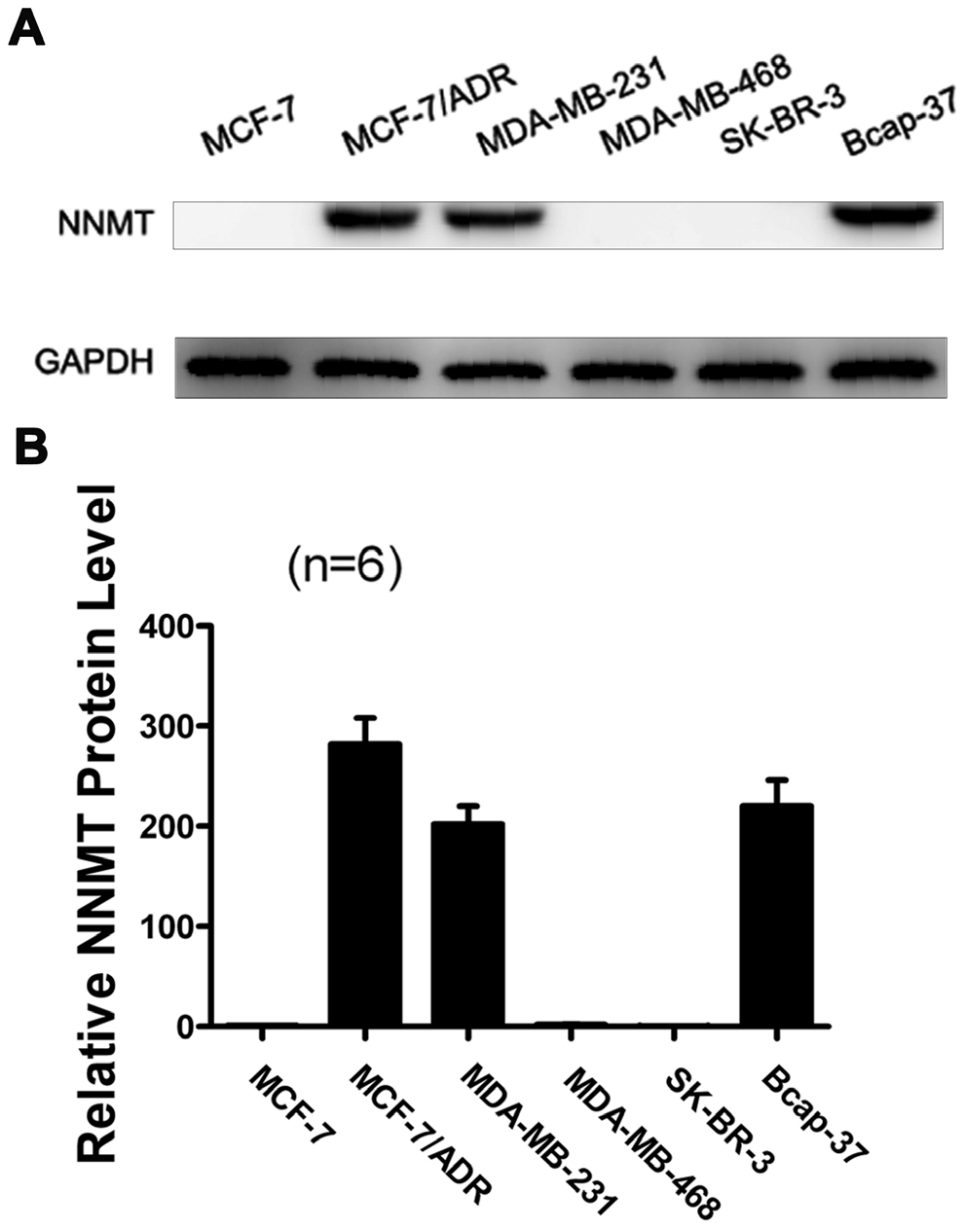
Expression of NNMT in breast cancer cells. Western blot was used to analyze NNMT protein expression levels in breast cancer cells. GAPDH was used as the internal control. High protein expression levels of NNMT were detected in MCF-7/ADR, MDA-MB-231 and Bcap-37. (A) The data are representative of at least three independent experiments of Western blot. (B) Protein quantification of the western blot results shown in (A). Protein levels are normalized to the GAPDH level and shown relative to the MCF-7 cells (normalized as 1).

To evaluate the NNMT expression in breast cancer cell lines, Western-blot was used to analyze protein and relative mRNA expression levels of NNMT. Not all the tested breast cancer cell lines were positive for expression of NNMT. Bcap-37, MDA-MB-231 and MCF-7/ADR showed high expression of NNMT, while MCF-7, SK-BR-3 and MDA-MB-468 showed either no or low expression (Figure 1). As MCF-7/ADR displays the highest level of NNMT protein expression, the knockdown experiment was performed on this cell line, and the inhibition of cell growth and increased apoptosis percentage were also found (refer to Figure S1). However, MCF-7/ADR is not a natural cell line which grows poorly without doxorubicin. While some other studies indicated that MDA-MB-468 breast cancer cells could express NNMT [28], but we did not confirm this point in our study. Therefore, we did not use MCF-7/ADR and MDA-MB-468 cell lines to perform the further experiment. To study the effect of down-regulation or overexpression of NNMT on cell biological process, Bcap-37 and MDA-MB-231; MCF-7 and SK-BR-3 cell lines were selected for further study, respectively.

### Down-regulation of NNMT expression inhibited the Cell Growth *in vitro* and *in vitro*

**Figure 2:**
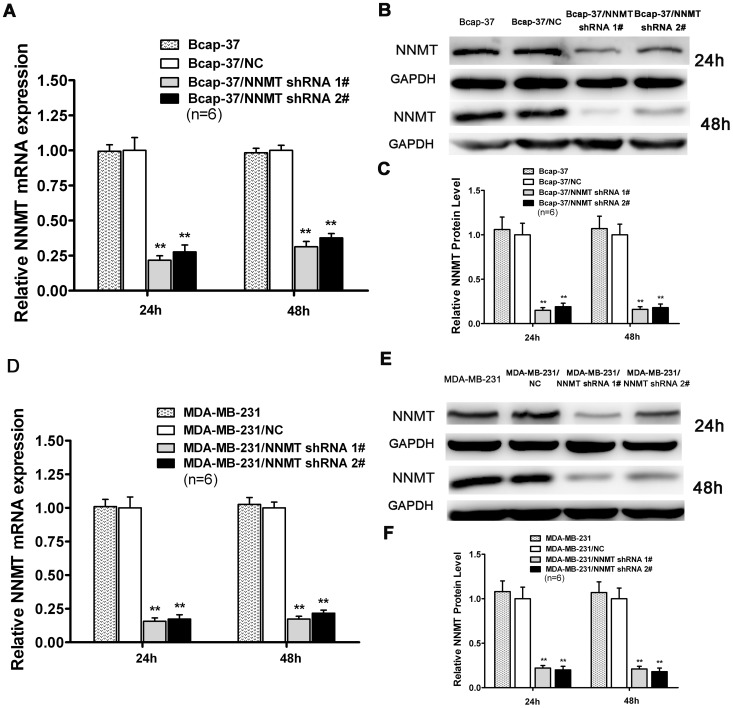
Lentiviral shRNA-NNMT efficaciously down-regulated expression of NNMT. Real-Time RT-PCR analysis (A, D) and Western blot (B, C, E, F) were used to analyze NNMT expression in Bcap-37, Bcap-37/NC, Bcap-37/NNMT shRNA 1#, Bcap-37/NNMT shRNA 2#, MDA-MB-231, MDA-MB-231/NC, MDA-MB-231/NNMT shRNA 1# and MDA-MB-231/NNMT shRNA 2# cells after infected for 24 h and 48 h. GAPDH was used as an internal control. (A, B, C) NNMT mRNA and protein levels were reduced significantly after infected with NNMT shRNA 1# and NNMT shRNA 2# in Bcap-37 cells compared to the Bcap-37/NC. (D, E, F) NNMT mRNA and protein levels were reduced significantly after infected with NNMT shRNA 1# and NNMT shRNA 2# in MDA-MB-231 cells compared to the MDA-MB-231/NC. (C) and (F) shows the protein quantification of the Western blot results shown in (B) and (E), respectively. The mRNA and protein levels were normalized to GAPDH level and are shown relative to the control groups (normalized as 1). Values in (A, C, D, F) are expressed as means ± SD of six independent experiments. There was no statistical significance between cells infected with shRNA NC and wild type cells both in Bcap-37 cells and MDA-MB-231 cells. No significant difference of effectiveness was found between shRNA#1 and shRNA#2 in MDA-MB-231 cells. **P<0.01 vs. NC.

The efficacy in down-regulating expression of NNMT gene was detected by real-time quantitative RT-PCR and Western blot (Figure 2). Compared with shRNA NC, mRNA and protein levels of NNMT were reduced significantly after NNMT shRNA 1# and NNMT shRNA 2# lentivirus infected into Bcap-37 and MDA-MB-231 cell lines (*p*<0.01). There was no statistical significance between cells infected with shRNA NC and wild type cells both in Bcap-37 cells and MDA-MB-231 cells.

**Figure 3:**
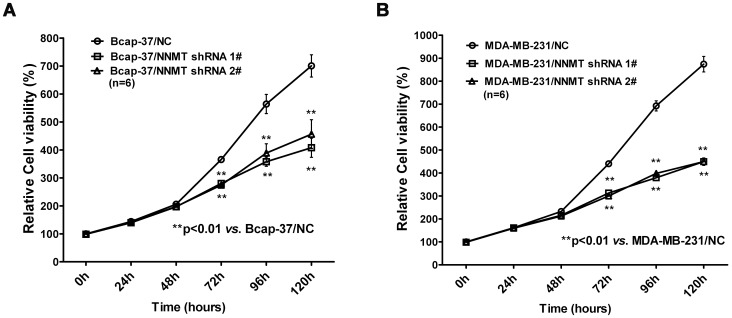
Down-regulation of NNMT expression inhibited the cell growth *in vitro*. (A, B) Cell growth was analyzed using the MTT assay. As shown in (A), remarkably low proliferation rates were observed in Bcap-37/NNMT shRNA 1# and Bcap-37/NNMT shRNA 2# cells compared to Bcap-37/NC cells after 72 h after seeding the cells in plates; the similar results were found in MDA-MB-231 cell models (B). The absorbance values at each time point were compared to that of control group at 0 h, which was normalized as 100%. Values are expressed as means ± SD of six independent experiments. **P<0.01 vs. NC.

To evaluate the effect of NNMT specific shRNAs on cell growth *in vitro*, colorimetric MTT assay was conducted. As shown in Figure 3A and 3B, efficient down-regulation of NNMT resulted in markedly reduced breast cancer cell growth both in Bcap-37 and MDA-MB-231 cell lines. A significant reduction of cell growth could be detected from 72 h when compared to the control cells (*p*<0.01). These results indicated that NNMT specific shRNAs attenuated the cell growth of breast cancer cells.

**Figure 4:**
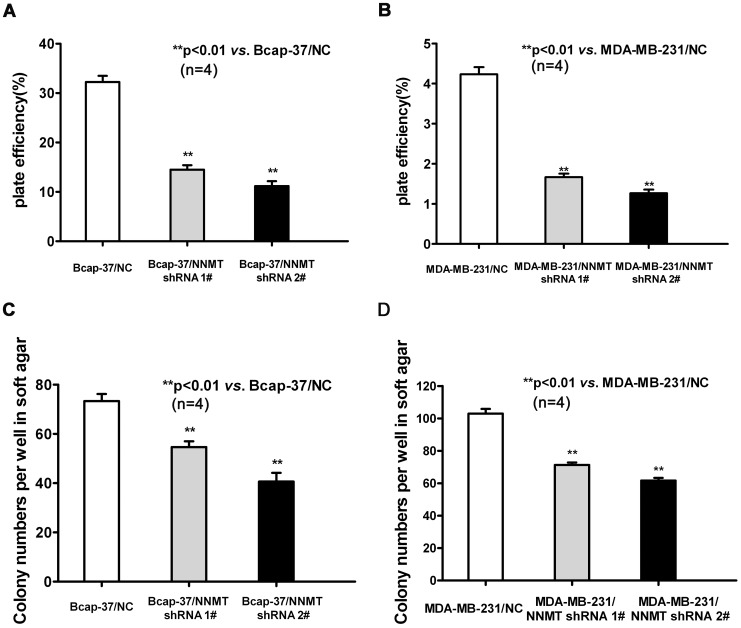
Down-regulation of NNMT expression decreased the plate efficiency and attenuated the capacity of colony formation in soft ager (A, B) To test plate colony formation of Bcap-37 and MDA-MB-231 cells infected with NNMT shRNAs, cells were placed in wells with media and incubated for 14 days before counting the number of colonies (foci>50 µm). The plate efficiency of Bcap-37/NNMT shRNA 1# and Bcap-37/NNMT shRNA 2# cells were lower than that of Bcap-37/NC group. The similar result was found in MDA-MB-231 cell models (B). (C, D) Colony formation of Bcap-37 and MDA-MB-231 infected with NNMT shRNAs was carried out by placing cells in media containing soft ager for 14 days. The numbers of Bcap-37/NNMT shRNA 1# and Bcap-37/NNMT shRNA foci >100 µm were less than that of Bcap-37/NC group (**P<0.01). The similar result was found in MDA-MB-231 cell models (D). Values are expressed as means ± SD of four independent experiments.

Plate colony formation and soft agar colony formation assay were conducted to validate the changes in cell proliferation observed using the MTT assay. The plate efficiency of Bcap-37/NNMT shRNA 1# and Bcap-37/NNMT shRNA 2# cells were lower than that of Bcap-37/NC group (Figure 4A). The similar result was found in MDA-MB-231 cell models (Figure 4B). The results of soft agar colony formation assay indicated efficient down-regulation of NNMT attenuates the capacity of colony formation (Figure 4C and 4D) (*p*<0.01).

**Figure 5:**
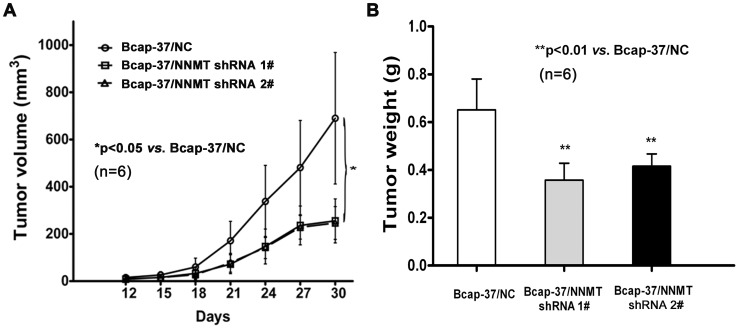
Down-regulation of NNMT expression inhibited the tumor growth *in vivo* (A, B) Xenograft experiment was used to assess the effect of down-regulation of NNMT expression on the cell growth of Bcap-37 *in vivo* (n = 6 for each group). Mice in all groups developed tumors. (A) The xenograft tumor volume was measured using calipers every three days. The average xenograft tumor volume was significantly smaller in Bcap-37 cells infected with NNMT shRNAs (NNMT shRNA 1# and NNMT shRNA 2#). (B) The average tumor weight was significantly lower in Bcap-37 cells infected with NNMT shRNAs at day 30. Values are expressed as means ± SD. There was no statistical significance between cells infected with NNMT shRNA 1# and shRNA 2# (*P<0.05; **P<0.01).

To investigate the effect of NNMT knockdown on breast cancer cells growth *in vivo*, Bcap-37 cells infected with NNMT shRNAs (NNMT shRNA 1# and NNMT shRNA 2#) and shRNA NC were subcutaneously injected into the upper portion of the right hind limb of 6 BALB/c nude mice for each group separately. Mice in all the groups developed tumors. After 30 days, the mean size of tumors developing in mice injected with infected NNMT shRNA 1# (255.4±93.1 mm^3^) and NNMT shRNA 2# (246.4±69.4 mm^3^) cells were significantly lower than that in mice injected with infected shRNA NC cells (690.3±278.4 mm^3^, n = 6) (*p*<0.05, Figure 5A). The similar result was found in tumor weight. The mean weight of tumors of Bcap-37/NNMT shRNA 1# (0.36±0.07 g, n = 6) and Bcap-37/NNMT shRNA 2# (0.42±0.05 g, n = 6) groups were significantly lower than Bcap-37/NC groups (0.65±0.13 g, n = 6) (*p*<0.01, Figure 5B). These results indicated shRNA-mediated NNMT knockdown suppresses tumorigenicity in Bcap-37 cells. Taken together, down-regulation of NNMT expression inhibited the cell growth *in vitro* and *in vivo*.

### Down-regulation of NNMT expression increased apoptosis and the ratio of Bax/Bcl-2

**Figure 6:**
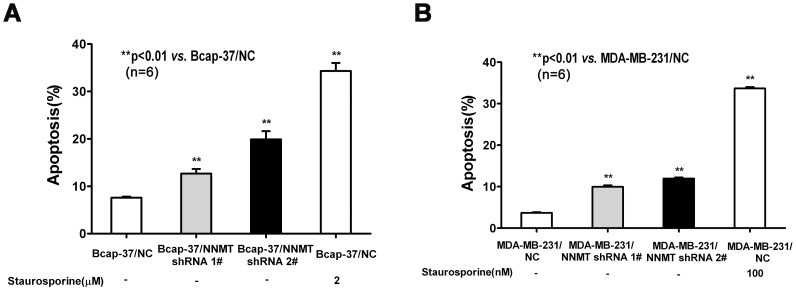
Down-regulation of NNMT expression induced apoptosis. Apoptosis was detected by flow cytometric analysis using the Annexin V-PE/7-AAD Apoptosis Detection Kit after infected for 48 h. (A) Bcap-37 cells were treated with NNMT shRNAs; (B) MDA-MB-231 cells were treated with NNMT shRNAs. The extent of apoptosis is expressed as the sum total percentages of annexin-positive populations. The percentage of apoptosis populations was increased in both cell lines infected with NNMT shRNA 1# and shRNA 2# compared to negative control cells. Cells treated with staurosporine were used as a positive control. Values are expressed as means ± SD of six independent experiments. The apoptosis of cells infected with NNMT shRNA 2# was higher than those infected with NNMT shRNA 1# both in Bcap-37 cells and MDA-MB-231 cells. **P<0.01 vs. NC.

To determine the role of NNMT in cancer cell survival, MDA-MB-231 and Bcap-37 cells were treated with NNMT shRNAs and subsequently flow cytometry was used to quantify apoptosis. The extent of apoptosis was expressed as the sum of total percentages of annexin-positive populations, which represented the early apoptosis (Annexin V-PE positive/7-AAD negative) and late apoptosis (Annexin V-PE positive/7-AAD positive). As shown in Figure 6A, the apoptosis of Bcap-37/NNMT shRNA 1# cells (12.7±1.0%) and Bcap-37/NNMT shRNA 2# (19.9±1.7%) were significantly higher than Bcap-37/NC (7.6±0.2%) (*p*<0.01). The similar result was found in MDA-MB-231 cell models. The apoptosis of MDA-MB-231/NNMT shRNA 1# cells (10.0±0.6%) and MDA-MB-231/NNMT shRNA 2# (11.9±0.5%) were significantly higher than MDA-MB-231/NC (3.7±0.3%) (*p*<0.01, Figure 6B). These results indicated down-regulation of NNMT increased apoptosis in both cell lines infected with NNMT shRNA 1# and shRNA 2# compared to negative control cells.

**Figure 7:**
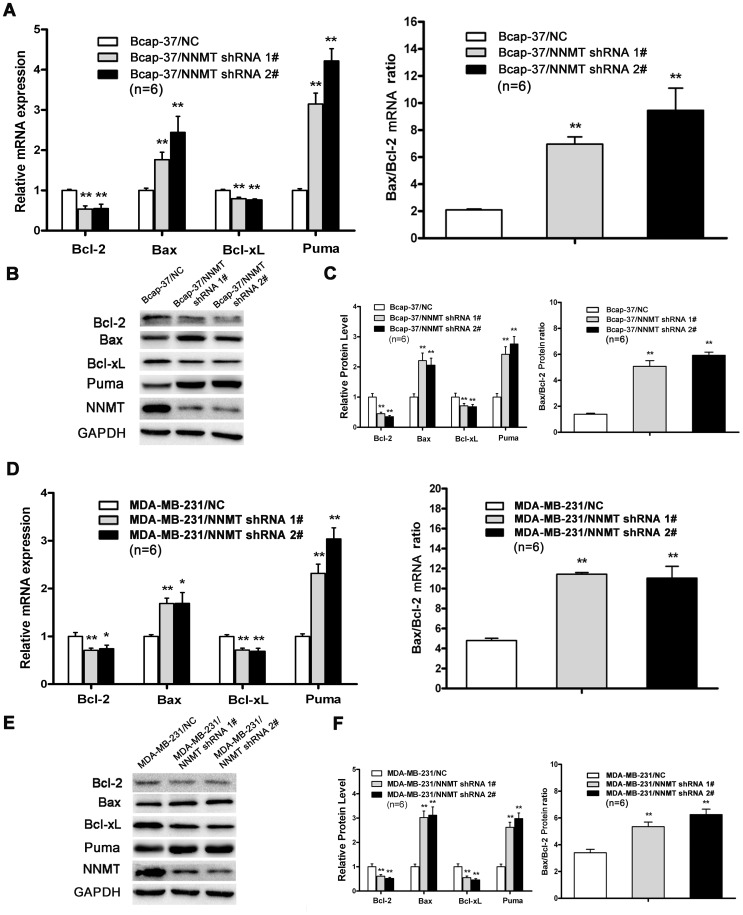
Construction of MCF-7 and SK-BR-3 cell strains expressing NNMT stably. Real-Time RT-PCR analysis (A, C) and Western blot (B, D) were used to analyze NNMT expression in MCF-7, MCF-7/Vector, MCF-7/NNMT-1, MCF-7/NNMT-2, SK-BR-3, SK-BR-3/Vector, SK-BR-3/NNMT-1 and SK-BR-3/NNMT-2. GAPDH was used as an internal control. (A, B) NNMT mRNA and protein levels were increased significantly after transfected with pcDNA3.1/NNMT in MCF-7 cells compared to the MCF-7/Vector. (C, D) NNMT mRNA and protein levels were increased significantly after transfected with pcDNA3.1/NNMT in SK-BR-3 cells compared to the SK-BR-3/Vector. The differences between cells transfected with pcDNA3.1 and wild type cells were not significant both in MCF-7 cells and SK-BR-3 cells. (B) and (D) shows the protein quantification of the western blot results, respectively. The mRNA and protein levels were normalized to GAPDH level and are shown relative to the control groups (normalized as 1). Values in (B, D) are expressed as means ± SD of four independent experiments. *P<0.05 vs. control group.

The Bcl-2 family of proteins, which shares homology in any of the four common Bcl-2 homology (BH) domains, was highly related with apoptosis [29]. Thus, we analyzed the changes of Bcl-2 family and found that the expression of Bax, Bcl-2, Bcl-xL and Puma significantly changed in NNMT down-regulated cells. As shown in Figure 7, the expression of pro-apoptotic proteins, Bax and Puma, was up-regulated significantly in both cell lines infected with NNMT shRNA 1# and NNMT shRNA 2# (*p*<0.01). On the contrary, the expression of Bcl-2 and Bcl-xL, which are identified as anti-apoptotic proteins, was significantly down-regulated (*p*<0.01). As a result, the down-regulation of NNMT increased both mRNA and protein ratio of Bax/Bcl-2 compared to negative control (*p*<0.01). This result indicated that apoptosis might be induced by down-regulation of NNMT via regulating the Bax/Bcl-2 ratio in human breast cancer cells.

### Overexpression of NNMT promoted cell growth and inhibited apoptosis

**Figure 8:**
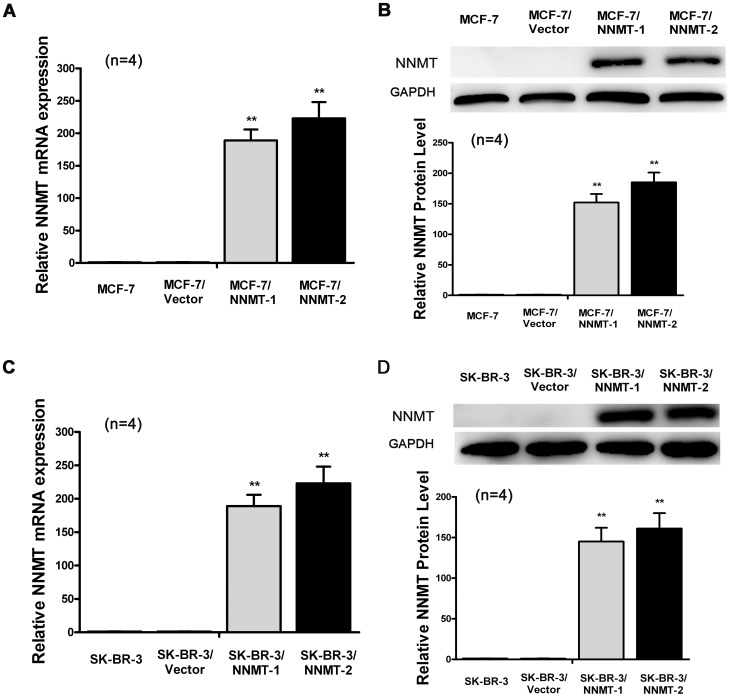
Overexpression of NNMT promoted the cell growth *in vitro*. (A, B) Cell growth was analyzed using the MTT assay. As shown in (A), higher proliferation rates were observed in MCF-7/NNMT-1 and MCF-7/NNMT-2 cells compared to MCF-7/Vector cells after 72 h after seeding the cells in plates; the similar results were found in SK-BR-3 cell models (B). The absorbance values at each time point were compared to that of control group at 0 h, which was normalized as 100%. Values are expressed as means ± SD of four independent experiments. **P<0.01 vs. control group; *P<0.05 vs. control group.

To confirm the effect of NNMT on cell proliferation and apoptosis, MCF-7 and SK-BR-3, which lack constitutive expression of NNMT, were transfected with pcDNA3.1/NNMT. MCF-7/NNMT-1, MCF-7/NNMT-2, SK-BR-3/NNMT-1 and SK-BR-3/NNMT-2, in which stable NNMT overexpression confirmed by real-time quantitative RT-PCR and Western blot, were selected for further analysis (Figure 8).

**Figure 9:**
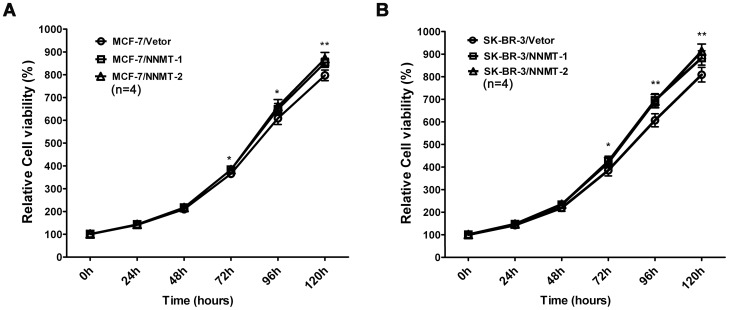
Overexpression of NNMT increased the plate efficiency and enhanced the capacity of colony formation in soft ager. (A, B) To test plate colony formation of MCF-7 and SK-BR-3 cells transfected with pcDNA3.1/NNMT, cells were placed in wells with media and incubated for 14 days before counting the number of colonies (foci>50 µm). The plate efficiency of MCF-7/NNMT-1 and MCF-7/NNMT-2 cells was higher than that of MCF-7/Vector group. The similar result was found in SK-BR-3 cell models (B). (C, D) The colony formation numbers of MCF-7/NNMT-1 and MCF-7/NNMT-2 cells foci >100 µm after 14 days were more numerous than that of MCF-7/Vector group (*P<0.05). The similar result was found in SK-BR-3 cell models (D). Values are expressed as means ± SD of four independent experiments.

**Figure 10:**
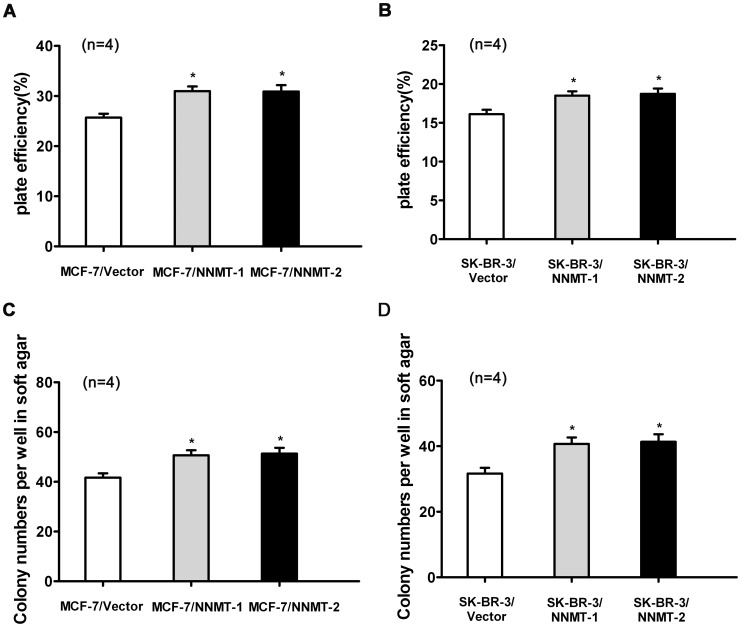
Overexpression of NNMT attenuated apoptosis. Apoptosis was detected by flow cytometric analysis using the Annexin V-PE/7-AAD Apoptosis Detection Kit after infected for 48 h. (A) MCF-7 cells were transfected with pcDNA3.1/NNMT; (B) SK-BR-3 cells were transfected with pcDNA3.1/NNMT. The percentage of apoptosis populations was decreased in both cell lines transfected with pcDNA3.1/NNMT compared to control cells. Cells treated with staurosporine were used as a positive control. Values are expressed as means ± SD of four independent experiments. **P<0.01 vs. control group; *P<0.05 vs. control group.

And as shown in Figure 9, overexpression of NNMT resulted in increased breast cancer cell growth both in MCF-7 and SK-BR-3 cell lines using MTT assay. A significant higher proliferation rate could be detected from 72 h when compared to the control cells (*p*<0.05). Consistent with the results of MTT assay, the colonies formed by NNMT-overexpressed cells were more numerous than those formed by control group (*p*<0.05, Figure 10).

**Figure 11:**
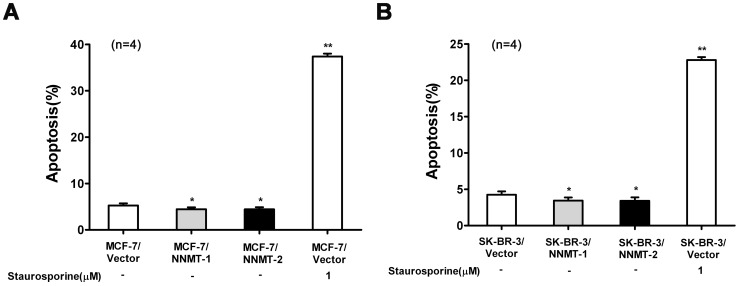
Effect of Down-regulation of NNMT expression on Bcl-2 family proteins. The expression levels of pro-apoptotic genes (Bax, Puma) and anti-apoptotic genes (Bcl-2, Bcl-xL) mRNA (A, D) and protein (B, C, E, F) were analyzed after infected for 48 h by real-time RT-PCR and Western blot, respectively. The expression of Bax and Puma was up-regulated significantly while the expression of Bcl-2 and Bcl-xL significantly down-regulated in both cell lines infected with NNMT shRNA 1# and shRNA 2# compared to negative control cells (p<0.01). As a result, both mRNA and protein ratio of Bax/Bcl-2 increased in both cell lines (*p*<0.01). GAPDH was used as an internal control. The data of (B) and (E) are representative of four independent experiments of Western blot. (C) and (F) shows the protein quantification of the western blot results shown in (B) and (E), respectively. The mRNA and protein levels were normalized to GAPDH level and all values were shown compared to the NC, which was normalized as 1. The ratio for Bax/Bcl-2 mRNA and protein was reading as compared to GAPDH. Values in (A, C, D, F) are expressed as means ± SD of six independent experiments. *P<0.05 vs. NC; **P<0.01 vs. NC.

To confirm the role of NNMT in cancer cell survival, the effect of NNMT overexpression on apoptosis was also quantified by flow cytometry. The data showed that overexpression of NNMT attenuates apoptosis in both NNMT overexpressed cell lines compared to negative control cells (*p*<0.05, Figure 11).

All these results showed that overexpression of NNMT could increase cell growth, tumorigenicity and inhibit cell apoptosis, which were consistent with the function of NNMT indicated by down-regulation of NNMT.

### Down-regulation of NNMT expression increased ROS production

**Figure 12:**
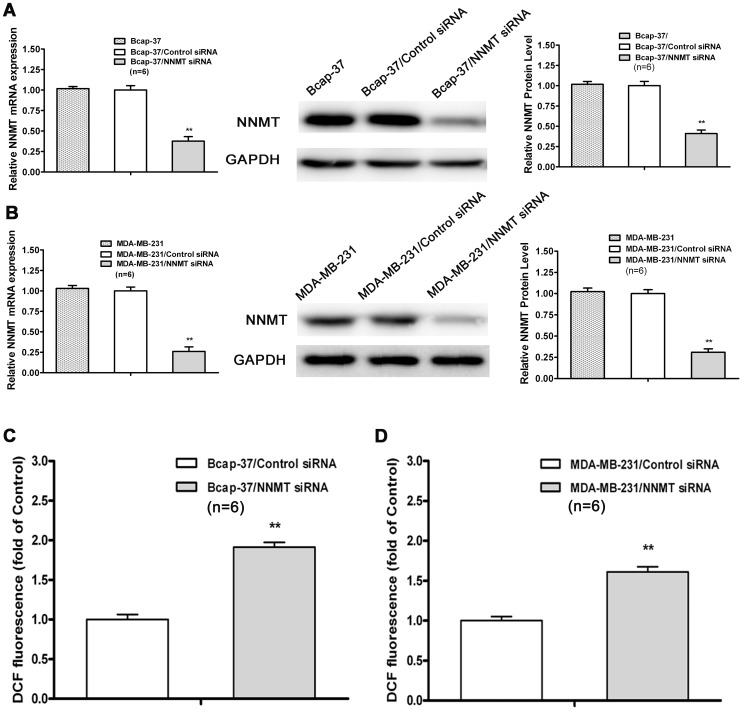
Down-regulation of NNMT expression increased ROS production. (A, B) The expression of NNMT in Bcap-37 and MDA-MB-231 cells transfected by siRNAs at a final concentration of 80 nM was analyzed by real-time RT-PCR and Western blot. GAPDH was used as an internal control. The mRNA and protein levels were normalized to GAPDH level and all values were shown compared to the NC, which was normalized as 1. Values are expressed as means ± SD of six independent experiments. There was statistical significance between cells transfected with NNMT siRNAs groups and NC groups (**P<0.01), while there was no significant difference between wild type cells and cells transfected with control siRNA. (C, D) Intracellular ROS was detected by flow cytometry using the fluorescent probe 2′,7′-dichlorodihydrofluorescein diacetate. Results are represented as fold of DCF fluorescence compared to control. ROS increased significantly in both cell lines of Bcap-37 and MDA-MB-231 treated with NNMT siRNA (**P<0.01). Values are expressed as means ± SD of six independent experiments.

ROS production was associated with apoptosis [30] and increasing intracellular ROS levels was highly related to apoptosis induction [31]. The mitochondria-mediated apoptotic pathway of cell death is especially susceptible to ROS. To assess the ROS production in NNMT knockdown breast cancer cells, the NNMT specific siRNAs, instead of shRNAs, was used to avoid interference of fluorescence of GFP in lentiviral vector. The efficacy in down-regulated expression of NNMT gene by siRNA was confirmed by real-time quantitative RT-PCR and Western blot (*p*<0.01, Figure 12A and 12B). And as shown in Figure 12C and 12D, down-regulation of NNMT significantly increased ROS production in both of Bcap-37 and MDA-MB-231 cell lines transfected with NNMT siRNAs (*p*<0.01).

### Down-regulation of NNMT expression activated the mitochondria-mediated apoptotic pathway

**Figure 13:**
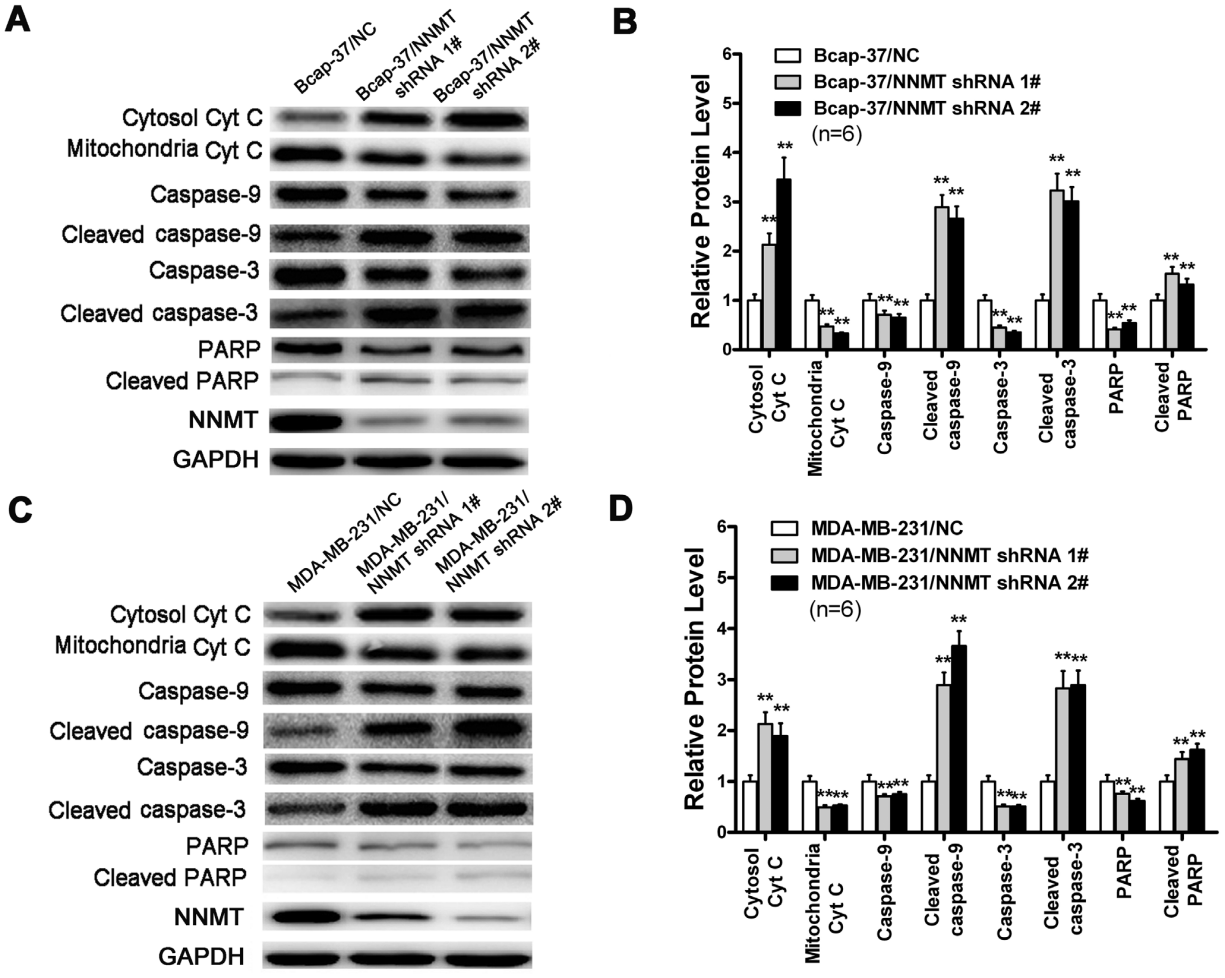
Effect of down-regulated NNMT on cytochrome c releasing and caspase processing. Cytochrome c in cytosolic and mitochondrial extracts and the processing of caspases-9, 3 and PARP in Bcap-37 cell (A) and MDA-MB-231 cell (C) were analyzed by Western blot. GAPDH was used as an internal control to ensure that equal amounts of proteins were loaded in each lane. (B) and (D) shows the protein quantification of the western blot results shown in (A) and (C), respectively. Cyt c in cytosolic compartment was significantly increased, while the amount of mitochondrial Cyt c was significantly decreased in both cell lines infected with NNMT shRNA 1# and shRNA 2# compared to negative control. In addition, the caspase-9, caspase-3 and PARP were significantly decreased, while the cleaved ones were found significantly increased compared to negative control. The protein levels were normalized to GAPDH level and all values were shown compared to the NC, which was normalized as 1. Values in (B, D) are expressed as means ± SD of six independent experiments. **P<0.01 vs. NC.

Down-regulation of NNMT increased mRNA and protein ratio of Bax/Bcl-2 and the production of intracellular ROS, which suggested that down-regulation of NNMT may be involved in mitochondria-mediated pathway. To determine whether down-regulation of NNMT induces apoptosis via the mitochondria-mediated pathway, we detected the release of Cyt c and the activation of related caspases, such as caspase-9 and caspase-3, which were key events in the mitochondria-mediated apoptotic pathway. As shown in Figure 13, Cyt c was observed to accumulate in cytosolic compartment in NNMT down-regulated Bcap-37 and MDA-MB-231 cells, while the amount of mitochondrial Cyt c was obviously decreased. In addition, the caspase-9, caspase-3 and PARP were decreased in NNMT knockdown Bcap-37 and MDA-MB-231 cells, while the cleaved ones were found significantly increased. Taken together, these results indicated that the down-regulation of NNMT in breast cancer cells resulted in activation of the mitochondria-mediated apoptotic pathway.

### Down-regulation of NNMT expression inactivated Akt and ERK1/2

**Figure 14:**
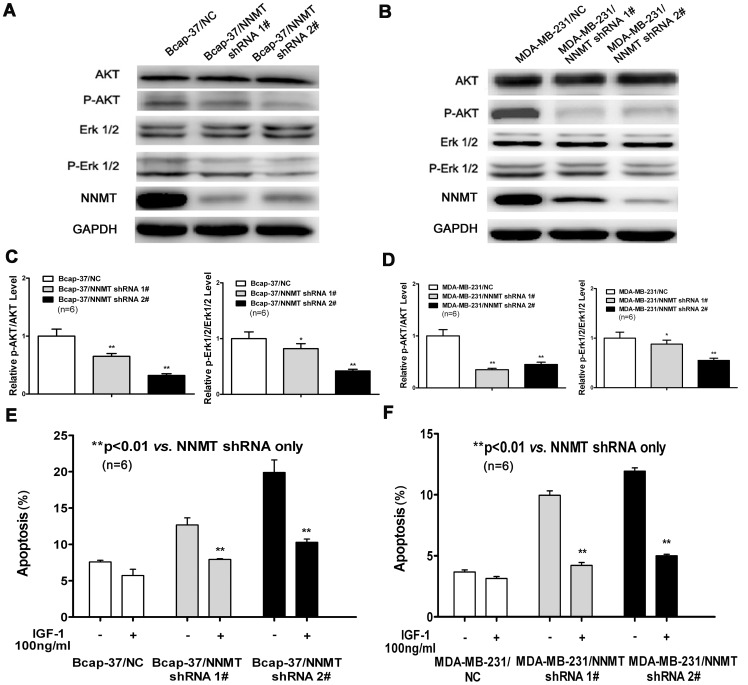
Down-regulation of NNMT expression inactivated Akt and ERK1/2. (A, B, C, D) The expression of Akt, p-Akt, ERK1/2 and p-ERK1/2 in Bcap-37 and MDA-MB-231 cells were analyzed by Western blot. GAPDH was used as an internal control. (C) and (D) show the ratio of p-AKT/AKT and p-ERK1/2/ERK1/2 after protein quantification of the western blot results shown in (A) and (B), respectively. The protein levels were normalized to GAPDH level and all values were shown compared to the NC, which was normalized as 1. Values in (C, D) are expressed as means ± SD of six independent experiments. The phosphorylation of Akt and ERK 1/2 was decreased significantly in both cell lines infected with NNMT shRNA 1# and shRNA 2# compared to negative control (*P<0.05; **P<0.01). (E, F) Effects of IGF-1 on apoptosis induced by down-regulation of NNMT were detected by Annexin V-PE and 7-AAD staining method. Each group of cells were seeded into 6-well plates with or without IGF-1 at a final concentration of 100 ng/ml. Apoptotic cells population was determined after incubation for 48 h. 100 ng/ml IGF-1 decreased the apoptosis significantly in NNMT knockdown Bcap-37 and MDA-MB-231 cells compared to negative control. Values are expressed as means ± SD of six independent experiments. (**P<0.01).

PI3K/Akt and MAPK pathways are the well known signaling cascade which participated in the regulation of cell progression and survival via protein phosphorylation [32], [33]. We tested key signaling components in PI3K/Akt and MAPK pathways and found that the phosphorylation of Akt and ERK 1/2 was decreased in NNMT shRNA treated cells. As shown in Figure 14A, 14B, 14C and 14D, down-regulation of NNMT decreased the expression levels of p-Akt and p-ERK1/2 in Bcap-37 and MDA-MB-231 cells and also decreased the ratio of p-AKT/AKT and p-ERK1/2/ERK1/2. Furthermore, it was confirmed by IGF-1 (Sigma, St. Louis, MO, USA), a potent activator of PI3K/Akt. 100 ng/ml IGF-1 partially decreased the apoptosis in NNMT shRNA treated cells (Figure 14E and 14F), suggesting the Akt pathway involved in apoptosis induced by down-regulation of NNMT.

## Discussion

NNMT is predominantly expressed in liver, catalyzes the N-methylation of nicotinamide, pyridines, and other structural analogues that are involved in the biotransformation and detoxification of many drugs and xenobiotic compounds. It plays a pivotal role in cellular events by regulating nicotinamide balance such as energy production, longevity, and cellular resistance to stress or injury [6], [34]–[36]. To the best of our knowledge, there is no direct study on the biological process of NNMT in breast cancer up to now. The only public correlation of NNMT and breast cancer was that NNMT had been found over-expressed in adriamycin-resistant breast cancer cell line MCF-7/ADR compared with its parent cell line MCF-7 [37]. We confirmed high expression of NNMT in some of the breast cancer cell lines, however, the role of NNMT in breast cancer is largely unknown.

In the present study, we investigated the biological function of NNMT in breast cancer cell lines (Bcap-37 and MDA-MB-231). ShRNA lentiviral vector against NNMT was designed to inhibit endogenous NNMT expression in both cell lines. Accompany with the down-regulation of NNMT expression, a significant inhibition of cell growth of Bcap-37 and MDA-MB-231 cells was found. The results of nude mice tumorigenesis experiments on Bcap-37 also showed that down-regulation of NNMT expression inhibited cancer cells tumorigenicity *in vivo*. The silencing reciprocal effect of NNMT was confirmed by over-expressing NNMT in the MCF-7 and SK-BR-3 breast cancer cell lines which lack constitutive expression of NNMT. Our data are consistent with the results derived from bladder cancer cells [38], KB cancer cells [24], oral carcinoma cells [26] and renal cancer cells [25], which strongly suggested that NNMT plays an important role in cancer cell growth i*n vitro* and *in vivo*.

Defective apoptotic machinery often confers survival advantage of cancer cells [29], and apoptosis attenuation is important in progressing to states of high-grade malignancy and resistance to therapy in tumors [39], [40]. Thus,we analyzed the effect of down-regulation of NNMT on apoptosis. There was a higher percentage of apoptosis population in Bcap-37 and MDA-MB-231 cells infected with NNMT shRNA. The cleaved-caspase-3 and cleaved PARP, which are reliable markers of apoptosis, were also showed increased by down-regulation of NNMT. On the contrary, overexpression of NNMT in the MCF-7 and SK-BR-3 breast cancer cell lines showed attenuated apoptosis when compared to negative control cells. Those results together demonstrated that down-regulation of NNMT induces apoptosis in Bcap-37 and MDA-MB-231, which also suppose that NNMT may play a vital role in breast cancer development via apoptosis. The underlying molecular mechanisms of the apoptosis promoted by down-regulation of NNMT in breast cancer cells would further clear the role of NNMT in cancer cells.

The Bcl-2 family of proteins, main apoptosis regulators, was designed to explain the mechanism of apoptosis induced by down-regulation of NNMT. In the present study, we observed that the expression of Bax and Puma was up-regulated, while the expression of Bcl-2 and Bcl-xL was significantly down-regulated in NNMT shRNA infected breast cancer cells, which resulted in the increase of the ratio of Bax/Bcl-2. Among the Bcl-2 family members, anti-apoptotic Bcl-2 and Bcl-xL have been reported to protect the cells by interacting with mitochondrial proteins such as the adenine nucleotide translocase (ANT) or the voltage dependent anion channel (VDAC), thus preventing them from forming mitochondrial pores, protecting membrane integrity, and inhibiting the release of apoptogenic factors such as Cyt c [41]. On the contrary, Bax can homodimerize or heterodimerize with other pro-apoptotic members such as Bak or truncated Bid, disrupting the integrity of the outer mitochondrial membrane (OMM) by forming mitochondrial pores and increasing its permeability, which can then lead to the release of apoptogenic factors such as Cyt c [42]. Puma, a Bcl-2 family member acting as neutralizing anti-apoptotic proteins, can heterodimerize with Bcl-2 and Bcl-xL and sequester them, thereby blocking their anti-apoptotic action at the mitochondria [29]. Interestingly, down-regulation of NNMT increased ROS production in human breast cancer cell lines was found. It has been reported that increasing ROS production can damage mitochondrial membranes, leading to the opening of mitochondrial permeability transition pore (MPTP) and releasing Cyt c [43], [44]. Taken those results together, we infered that down-regulation of NNMT in human breast cancer may cause the mitochondria dysfunction and release of Cyt c from mitochondria. The ratio of Bax/Bcl-2 partially showed the response to proximal death and survival signals of cells as reported by Oltvai ZN, *et al* [45].

Cyt c plays a crucial role for the execution of the mitochondrial-mediated intrinsic pathway apoptosis because it can form apoptosome with apoptosis-activating factor 1(Apaf-1) and caspase-9 after releasing into the cytoplasm and activate the executioner caspases-3 and 7, which finally causes cell apoptosis through nuclear fragmentation of cells [46]–[49]. To confirm whether down-regulation of NNMT induces apoptosis via the mitochondria-mediated pathway, we analyzed the release of Cyt c and the activation of related caspases, such as caspase-9 and caspase-3, which were key events in the mitochondria-mediated apoptotic pathway. As expected, we have shown that Cyt c was released from mitochondrial fraction into cytosolic fraction and the cleaved caspase-9, caspase-3 and PARP were found significantly increased in NNMT shRNA infected cells. These results indicated that down-regulation of NNMT in breast cancer cells induces apoptosis via the mitochondria-mediated pathway by increasing the ratio of Bax/Bcl-2 and ROS production, resulting in releasing Cyt c from mitochondrial fraction into cytosolic to activate the executioner caspases-3 and 7.

In our study, we also found that the phosphorylation of Akt and ERK 1/2 was decreased in NNMT shRNA treated cells. Akt can inhibit apoptosis through multiple mechanisms and preventing AKT activation can induce apoptosis [50], [51]. The result of IGF-1 decreased the apoptosis in NNMT shRNA treated cells indicated that the apoptosis induced by down-regulation of NNMT can be attributed, at least partially to Akt inactivation. This also suggests that the Akt pathway involved in the effect of NNMT on cancer cells. Our results of down-regulation of NNMT on Akt pathway are in line with the most recent reports [52], [53]. Win KT, et al reported that NNMT overexpression was significantly positively associated with phosphorylation of Akt and indicated worse prognosis in patients with nasopharyngeal carcinoma recently [53]. Another report shown that NNMT expression regulates neurone morphology in vitro via the sequential activation of ephrin-B2 (EFNB2) and Akt cellular signaling pathways [52]. Diverse cellular functions, ranging from cell survival to cell death, are regulated by activation of ERK pathway [54]. We don’t know the exact mechanisms of How NNMT phosphorylates ERK and AKT in breast cancer so far. Ulanovskaya et al (Nat Chem Biol) recently reported that the methylation events regulated by NNMT can alter histone-dependent gene expression, but also extend beyond histones to include tumor suppressor proteins like PP2A. We suppose that the production of ROS and condition of PP2A methylation and demethylation regulated by NNMT may contribute to the phosphorylates ERK and AKT in breast cancer, however, this needs more detailed experiments to confirm.

In summary, we found that down-regulation of NNMT expression significantly inhibited cell growth, decreased tumorigenicity in mice and induced apoptosis via the mitochondria-mediated pathway. Although the definite mechanism of its role needs to be further studied, NNMT may become a promising candidate for breast cancer therapy.

## Supporting Information

Figure S1 Down-regulation of NNMT expression inhibited cell growth and induced apoptosis in MCF-7/ADR cells. (A) Western blot were used to analyze NNMT expression in MCF-7/ADR, MCF-7/ADR/NC, MCF-7/ADR/NNMT shRNA 1# and MCF-7/ADR/NNMT shRNA 2# after infected for 48 h. GAPDH was used as an internal control. (B) Cell growth was analyzed using the MTT assay. As shown, remarkably low proliferation rates were observed in MCF-7/ADR/NNMT shRNA 1# and MCF-7/ADR/NNMT shRNA 2# cells compared to MCF-7/ADR/NC cells after 72 h after seeding the cells in plates. The absorbance values at each time point were compared to that of control group at 0 h, which was normalized as 100%. Values are expressed as means ± SD of four independent experiments. (C) Apoptosis was detected by flow cytometric analysis using the Annexin V-PE/7-AAD Apoptosis Detection Kit after seeded for 48 h. The extent of apoptosis is expressed as the sum total percentages of annexin-positive populations. The percentage of apoptosis populations was increased in both cell lines infected with NNMT shRNA 1# and shRNA 2# compared to negative control cells. Values are expressed as means ± SD of four independent experiments. **P<0.01 vs. NC.
(TIF)
