# Correction: Down-Regulation of Nicotinamide N-methyltransferase Induces Apoptosis in Human Breast Cancer Cells via the Mitochondria-Mediated Pathway

**DOI:** 10.1371/journal.pone.0097721

**Published:** 2014-05-08

**Authors:** The PLOS ONE Staff

The figure legends for Figures 7-11 are incorrect. Please find the corrected legends and the related figures below.

**Figure 7:**
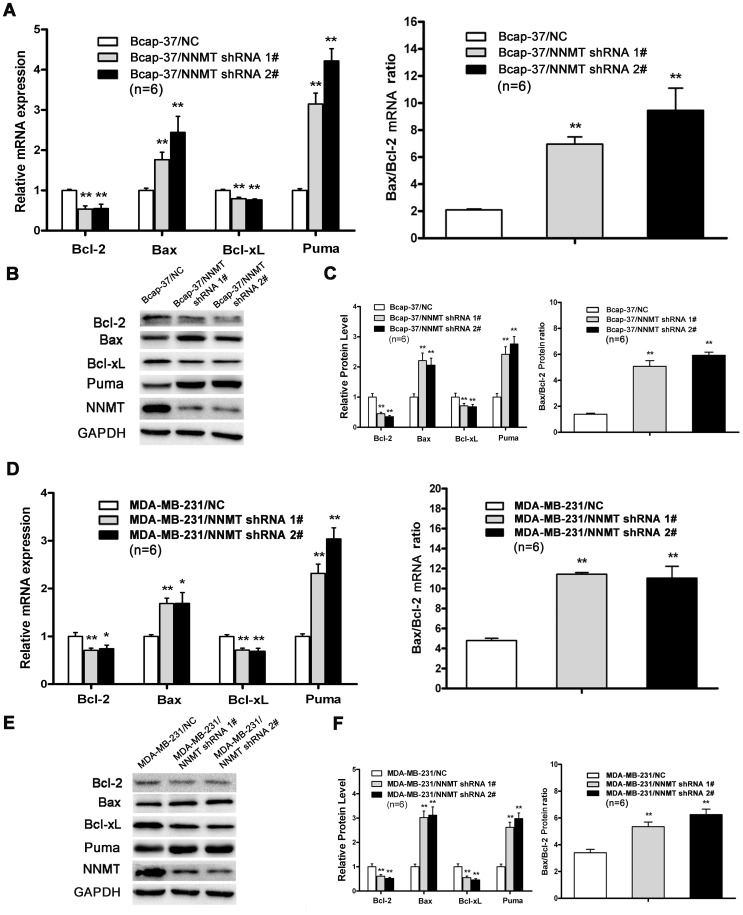
Effect of Down-regulation of NNMT expression on Bcl-2 family proteins. The expression levels of pro-apoptotic genes (Bax, Puma) and anti-apoptotic genes (Bcl-2, Bcl-xL) mRNA (A, D) and protein (B, C, E, F) were analyzed after infected for 48 h by real-time RT-PCR and Western blot, respectively. The expression of Bax and Puma was up-regulated significantly while the expression of Bcl-2 and Bcl-xL significantly down-regulated in both cell lines infected with NNMT shRNA 1# and shRNA 2# compared to negative control cells (p<0.01). As a result, both mRNA and protein ratio of Bax/Bcl-2 increased in both cell lines (*p*<0.01). GAPDH was used as an internal control. The data of (B) and (E) are representative of four independent experiments of Western blot. (C) and (F) shows the protein quantification of the western blot results shown in (B) and (E), respectively. The mRNA and protein levels were normalized to GAPDH level and all values were shown compared to the NC, which was normalized as 1. The ratio for Bax/Bcl-2 mRNA and protein was reading as compared to GAPDH. Values in (A, C, D, F) are expressed as means ± SD of six independent experiments. * *P* <0.05 vs. NC; **P<0.01 vs. NC.

**Figure 8:**
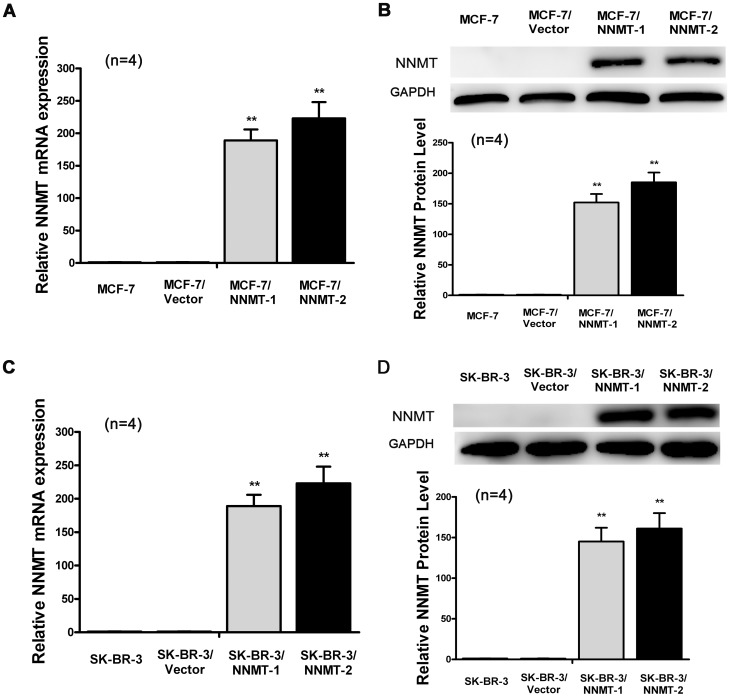
Construction of MCF-7 and SK-BR-3 cell strains expressing NNMT stably. Real-Time RT-PCR analysis (A, C) and Western blot (B, D) were used to analyze NNMT expression in MCF-7, MCF-7/Vector, MCF-7/NNMT-1, MCF-7/NNMT-2, SK-BR-3, SK-BR-3/Vector, SK-BR-3/NNMT-1 and SK-BR-3/NNMT-2. GAPDH was used as an internal control. (A, B) NNMT mRNA and protein levels were increased significantly after transfected with pcDNA3.1/NNMT in MCF-7 cells compared to the MCF-7/Vector. (C, D) NNMT mRNA and protein levels were increased significantly after transfected with pcDNA3.1/NNMT in SK-BR-3 cells compared to the SK-BR-3/Vector. The differences between cells transfected with pcDNA3.1 and wild type cells were not significant both in MCF-7 cells and SK-BR-3 cells. (B) and (D) shows the protein quantification of the western blot results, respectively. The mRNA and protein levels were normalized to GAPDH level and are shown relative to the control groups (normalized as 1). Values in (B, D) are expressed as means ± SD of four independent experiments. * *P* <0.05 vs. control group.

**Figure 9:**
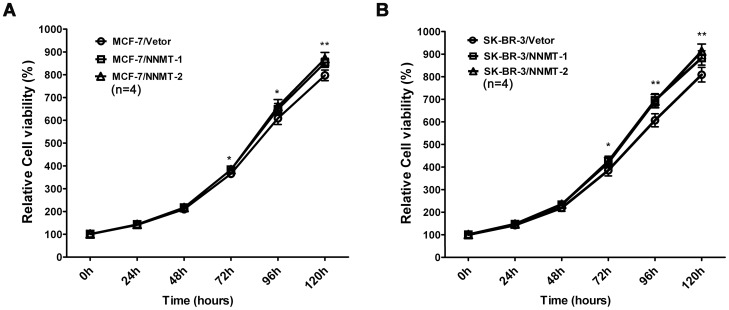
Overexpression of NNMT promoted the cell growth in vitro. (A, B) Cell growth was analyzed using the MTT assay. As shown in (A), higher proliferation rates were observed in MCF-7/NNMT-1 and MCF-7/NNMT-2 cells compared to MCF-7/Vector cells after 72 h after seeding the cells in plates; the similar results were found in SK-BR-3 cell models (B).The absorbance values at each time point were compared to that of control group at 0 h, which was normalized as 100%. Values are expressed as means ± SD of four independent experiments. ** *P* <0.01 vs. control group; * *P* <0.05 vs. control group.

**Figure 10:**
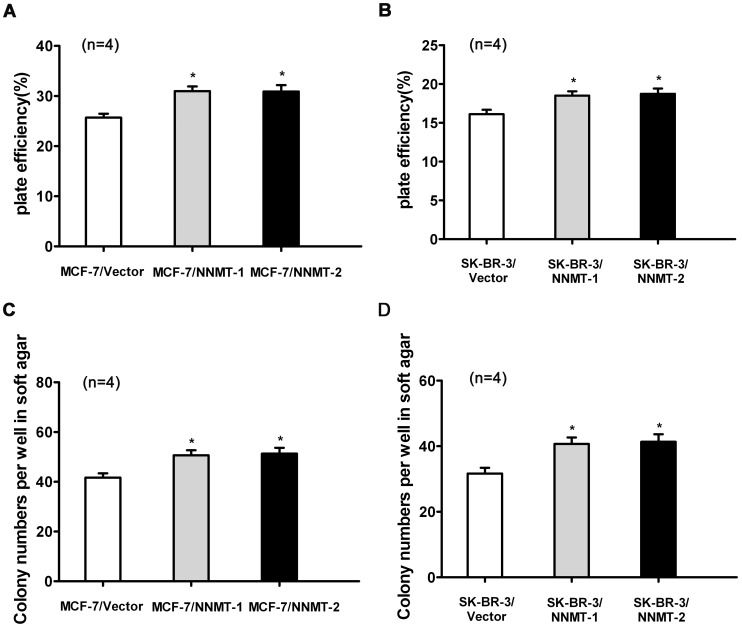
Overexpression of NNMT increased the plate efficiency and enhanced the capacity of colony formation in soft ager. (A, B) To test plate colony formation of MCF-7 and SK-BR-3 cells transfected with pcDNA3.1/NNMT, cells were placed in wells with media and incubated for 14 days before counting the number of colonies (foci>50µm). The plate efficiency of MCF-7/NNMT-1 and MCF-7/NNMT-2 cells was higher than that of MCF-7/Vector group. The similar result was found in SK-BR-3 cell models (B). (C, D) The colony formation numbers of MCF-7/NNMT-1 and MCF-7/NNMT-2 cells foci >100µm after 14 days were more numerous than that of MCF-7/Vector group (*P<0.05). The similar result was found in SK-BR-3 cell models (D). Values are expressed as means ± SD of four independent experiments.

**Figure 11:**
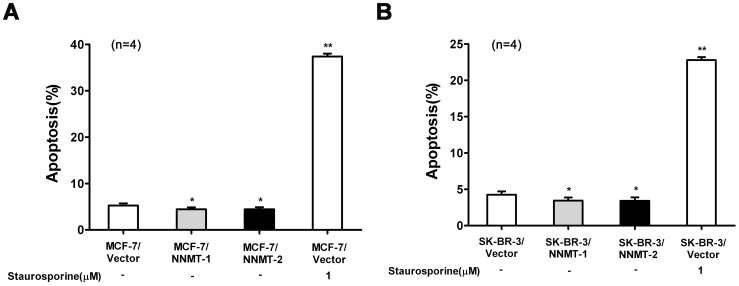
Overexpression of NNMT attenuated apoptosis. Apoptosis was detected by flow cytometric analysis using the Annexin V-PE/7-AAD Apoptosis Detection Kit after infected for 48 h. (A) MCF-7 cells were transfected with pcDNA3.1/NNMT; (B) SK-BR-3 cells were transfected with pcDNA3.1/NNMT. The percentage of apoptosis populations was decreased in both cell lines transfected with pcDNA3.1/NNMT compared to control cells. Cells treated with staurosporine were used as a positive control. Values are expressed as means ± SD of four independent experiments. ** *P* <0.01 vs. control group; * *P* <0.05 vs. control group.
