# Supplementary figures and images for: Down-Regulation of Nicotinamide N-methyltransferase Induces Apoptosis in Human Breast Cancer Cells via the Mitochondria-Mediated Pathway
Source: PLoS One. 2014 Feb 18;9(2):e89202. doi: 10.1371/journal.pone.0089202 (PMC3928407; doi:10.1371/journal.pone.0089202)

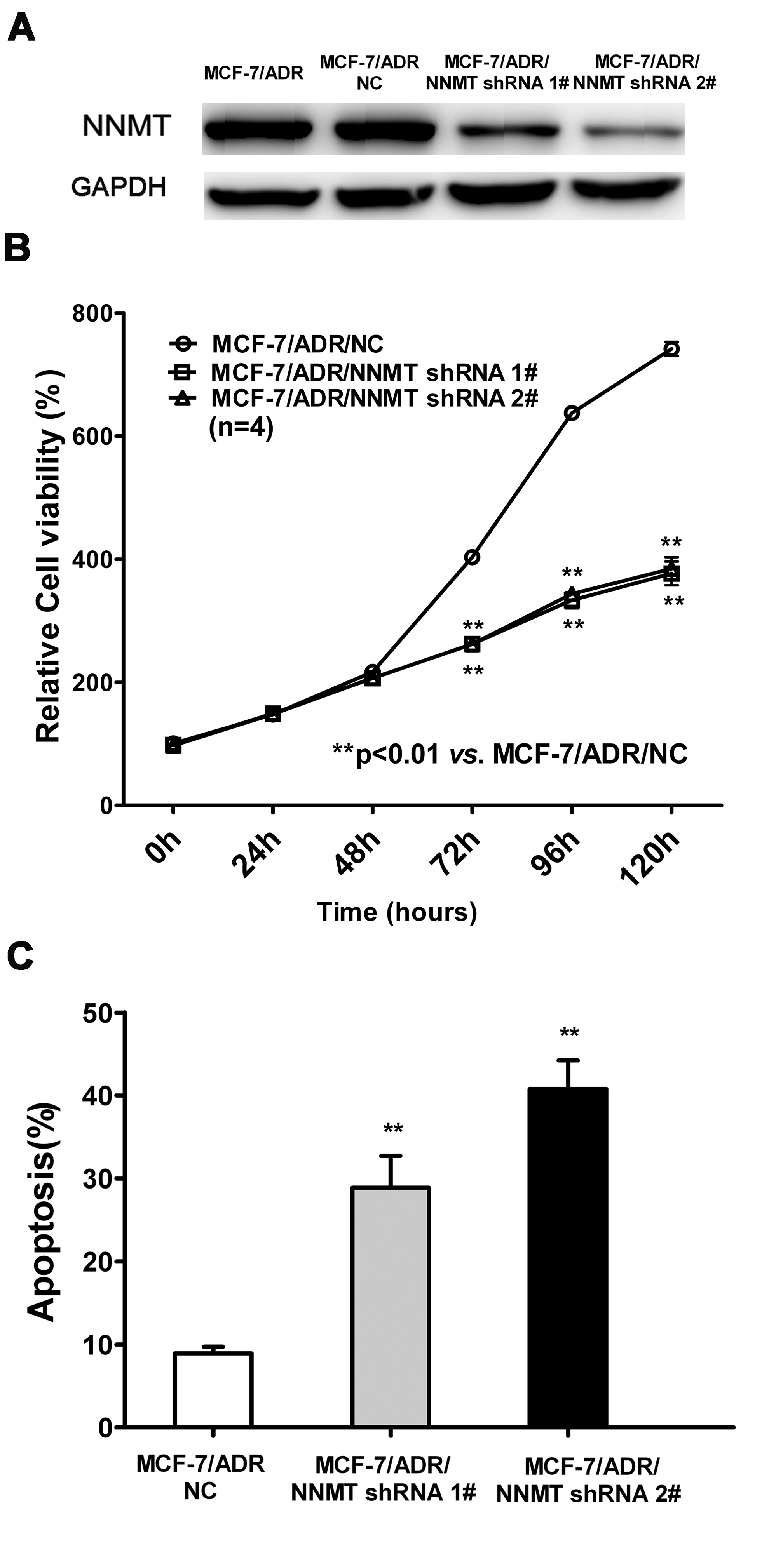

Supplement: Figure S1 — Down-regulation of NNMT expression inhibited cell growth and induced apoptosis in MCF-7/ADR cells. (A) Western blot were used to analyze NNMT expression in MCF-7/ADR, MCF-7/ADR/NC, MCF-7/ADR/NNMT shRNA 1# and MCF-7/ADR/NNMT shRNA 2# after infected for 48 h. GAPDH was used as an internal control. (B) Cell growth was analyzed using the MTT assay. As shown, remarkably low proliferation rates were observed in MCF-7/ADR/NNMT shRNA 1# and MCF-7/ADR/NNMT shRNA 2# cells compared to MCF-7/ADR/NC cells after 72 h after seeding the cells in plates. The absorbance values at each time point were compared to that of control group at 0 h, which was normalized as 100%. Values are expressed as means ± SD of four independent experiments. (C) Apoptosis was detected by flow cytometric analysis using the Annexin V-PE/7-AAD Apoptosis Detection Kit after seeded for 48 h. The extent of apoptosis is expressed as the sum total percentages of annexin-positive populations. The percentage of apoptosis populations was increased in both cell lines infected with NNMT shRNA 1# and shRNA 2# compared to negative control cells. Values are expressed as means ± SD of four independent experiments. **P<0.01 vs. NC. (TIF) [file pone.0089202.s001.tif]
